# Real-Time Home Automation System Using BCI Technology

**DOI:** 10.3390/biomimetics9100594

**Published:** 2024-10-01

**Authors:** Marius-Valentin Drăgoi, Ionuț Nisipeanu, Aurel Frimu, Ana-Maria Tălîngă, Anton Hadăr, Tiberiu Gabriel Dobrescu, Cosmin Petru Suciu, Andrei Rareș Manea

**Affiliations:** 1 Faculty of Engineering in Foreign Languages, National University of Science and Technology POLITEHNICA Bucharest, 060042 Bucharest, Romania; ionut.nisipeanu@stud.fils.upb.ro (I.N.); aurel_viorel.frimu@stud.fils.upb.ro (A.F.); 2 Department of Strength of Materials, Faculty of Industrial Engineering and Robotics, National University of Science and Technology POLITEHNICA Bucharest, 060042 Bucharest, Romania; anton.hadar@upb.ro; 3 Academy of Romanian Scientists, 3 Ilfov Street, Sector 5, 050045 Bucharest, Romania; 4 Technical Sciences Academy of Romania, 26 Dacia Boulevard, Sector 1, 030167 Bucharest, Romania; 5 Faculty of Industrial Engineering and Robotics, National University of Science and Technology POLITEHNICA Bucharest, 060042 Bucharest, Romania; tiberiu.dobrescu@upb.ro; 6 National Research and Development Institute for Gas Turbines COMOTI, 061126 Bucharest, Romania; cosmin.suciu@comoti.ro; 7 Faculty of Mechanical Engineering and Mechatronics, National University of Science and Technology POLITEHNICA Bucharest, 060042 Bucharest, Romania; andrei_rares.manea@stud.mec.upb.ro

**Keywords:** BCI, EEG, home security, Raspberry Pi, disabled people, biometric

## Abstract

A Brain–Computer Interface (BCI) processes and converts brain signals to provide commands to output devices to carry out certain tasks. The main purpose of BCIs is to replace or restore the missing or damaged functions of disabled people, including in neuromuscular disorders like Amyotrophic Lateral Sclerosis (ALS), cerebral palsy, stroke, or spinal cord injury. Hence, a BCI does not use neuromuscular output pathways; it bypasses traditional neuromuscular pathways by directly interpreting brain signals to command devices. Scientists have used several techniques like electroencephalography (EEG) and intracortical and electrocorticographic (ECoG) techniques to collect brain signals that are used to control robotic arms, prosthetics, wheelchairs, and several other devices. A non-invasive method of EEG is used for collecting and monitoring the signals of the brain. Implementing EEG-based BCI technology in home automation systems may facilitate a wide range of tasks for people with disabilities. It is important to assist and empower individuals with paralysis to engage with existing home automation systems and gadgets in this particular situation. This paper proposes a home security system to control a door and a light using an EEG-based BCI. The system prototype consists of the EMOTIV Insight™ headset, Raspberry Pi 4, a servo motor to open/close the door, and an LED. The system can be very helpful for disabled people, including arm amputees who cannot close or open doors or use a remote control to turn on or turn off lights. The system includes an application made in Flutter to receive notifications on a smartphone related to the status of the door and the LEDs. The disabled person can control the door as well as the LED using his/her brain signals detected by the EMOTIV Insight™ headset.

## 1. Introduction

Brain–Computer Interfaces (BCIs) assess brain signals and provide commands to output devices to carry out certain tasks. Brain–Computer Interfaces do not use neuromuscular output pathways [1]. 

The primary objective of BCIs is to substitute or reinstate functionality for those afflicted with neuromuscular conditions such as ALS, cerebral palsy, etc. [2,3]. Scientists have used electroencephalography and intracortical, electrocorticographic, and other brain signals to manipulate cursors, robotic legs, robotic arms, prosthetics, wheelchairs, TV remote controls, and several other devices since the first demonstrations of spelling and controlling individual neurons. BCIs have the potential to assist in the rehabilitation of individuals affected by stroke and other diseases. They have the potential to enhance the performance of surgeons and other medical professionals [4], since more than one billion people (about 15% of the global population) are disabled, and half of that group lacks the financial means to obtain adequate medical treatment, according to the World Health Organization (WHO) [5].

The rapid growth in research and development enterprises in BCI technology has generated enthusiasm among scientists, engineers, clinicians, and the public. Also, BCIs need signal acquisition technology that is both portable and dependable, ensuring safety and reliability in any situation. Additionally, it is crucial to develop practical and viable approaches for the widespread implementation of these technologies. BCI performance must provide consistent reliability on a daily and moment-to-moment basis to align with the normal functioning of muscles. The concept of incorporating sensors and intelligence into physical objects was first introduced in the 1980s by students from Carnegie Mellon University who modified a juice vending machine so that they could remotely monitor the contents of the machine [6].

In the last decade, an EEG-based BCI has been successfully used with Convolutional Neural Networking (CNN) to detect diseases like epilepsy [7]. EEG-based BCI technology has also been used to control a prosthetic lower limb [8,9] or a prosthetic upper limb [10]. It is expected that in the future, BCIs will be widespread in our lives, improving our way of living, especially for disabled people, who may accomplish different activities by speech imagery only [11]. 

A network of physical objects, autos, appliances, and other things that are fitted with sensors, software, and network connections is referred to as the Internet of Things (IoT) [12]. Because of this, they are able to collect and share information. Electronic devices, which are sometimes referred to as “smart objects”, refer to a wide range of technologies. These gadgets include simple smart home devices, including smart thermostats, wearable devices like smartwatches, and apparel with Radio Frequency Identification (RFID) technology, as well as complex industrial gear and transportation systems [13,14].

The Internet of Things (IoT) technology enables communication between internet-connected gadgets, as well as other devices such as smartphones and gateways. This leads to the formation of a vast interconnected system of devices that can autonomously exchange data and perform a diverse array of tasks. This includes a diverse array of applications, such as monitoring environmental conditions, improving traffic flow through the use of intelligent cars and other sophisticated automotive equipment, and tracking inventory and shipments in storage facilities, among others. For people with severe motor disabilities, having a smart home represents a necessity nowadays; they can not only manage daily used devices from home but will also be able to manage the security of the home [15].

During the past years, many approaches have been made to controlling a smart object or a software application by using EEG-based BCI signals. The following paragraphs present several related works that discuss the issue of BCI home automation and security.

In 2018, Qiang Gao et al. [16] proposed a safe and cost-effective online smart home system based on BCI to provide elderly and paralyzed people with a new supportive way to control home appliances. They used the Emotiv EPOC EEG headset to detect EEG signals, and these signals are denoised processed, and converted into commands. The system has the ability to identify several instructions for controlling four smart devices, including a web camera, a lamp, intelligent blinds, and a guardianship telephone. Additionally, they used Power over Ethernet (PoE) technology to provide both power and connectivity to these devices. The experimental results elucidated that their proposed system obtained an 86.88 ± 5.30% accuracy rate of average classification.

In 2020, K. Babu and P. Vardhini [17] implemented a system to control a software application, which can be used further in home automation. They used a NeuroSky headset, Arduino, an ATMEGA328P, and a laptop. A neurosoftware application was used to create three virtual objects represented by three icons and to control them by blinking using the headset user. Three ports from Arduino were dedicated to the three objects from the neuro application to simulate controlling a fan and a motor and to manage the switch between the fan and motor. The home appliance status was changed by running a MATLAB code.

Other experiments reveal an implemented prototype to control some home appliances like an LED and a fan, as in the implemented system presented by Lanka et al. [18]. They used a dedicated neural headset, a laptop, an ESP32 microcontroller, an LED, and a fan. Using Bluetooth technology, they connected the headset to the laptop, and this connected with the microcontroller. The fan and LED were wired and connected to the microcontroller. In this way, they developed a system to control a fan and an LED by a healthy, disabled, or paralysed user electric mind wave.

Eyhab Al-Masri et al. [19] published an article in 2022, where they specified the development of a BCI framework that helped people with motor disabilities to control Philips Hue smart lights and a Kasa Smart Plug using a dedicated neural headset. They used an EEG EMOTIV headset, a Raspberry Pi, a Kasa Smart Plug, and Philips Hue smart lights as hardware. Bluetooth technology is used to connect the headset to the Raspberry Pi. The commands are configured and transformed from the Raspberry Pi to the Kasa Smart Plug and Philips Hue smart lights using Node-RED. The experimental results showed the efficacy and practicability of using EEG signals to operate IoT devices with a precision rate of 95%.

In 2023, Danish Ahmed et al. [20] successfully used BCI technology to control a light and a fan via a dedicated neural headset. The implemented system consists of an EMOTIV EPOC headset, a PC laptop, an Arduino platform, and a box that contains a light and a fan. The headset is connected to the PC via Bluetooth, the laptop uses a WebSocket server and the JSON-RPC protocol to connect to Arduino, and Arduino is wired to the light and fan. The user trained the headset to control the prototype by his/her thoughts.

A new challenge has been overcome in home automation, which is controlling a TV using brainwaves. Several papers have discussed this issue. One of the systems was presented in 2023 by Haider Abdullah et al. [21], where they successfully implemented and tested this system on 20 participants. The proposed system includes the following components: an EMOTIV Insight headset, laptop (connected via Bluetooth with the headset), Raspberry Pi 4 (connected through SSH to the laptop), and TV remote control circuit (connected with wires to the Raspberry Pi). Three different brands of TVs were used in the system testing: SONY^®^, SHOWINC^®^, and SAMIX^®^. Four controlling commands were included in this EEG-based TV remote control: switching the TV on/off, volume changing, and channel changing. The test showed promising results, where the system’s accuracy was almost 74.9%.

The use of BCI technology for controlling different devices represents the new direction of advancement in both hardware and software development. In this context, this paper presents the design and implementation of a proposed real-time BCI-IoT system used to assure home security using a dedicated neuronal headset to control door-locking and light-using speech imagery. The proposed system enables disabled and paralysed people to lock or unlock a door and to turn an LED ON/OFF, with the ability to receive status notifications. The proposed system has been tested on twenty participants. The proposed system has been simulated using a Unity engine, as well as receiving a hardware implementation using a Raspberry Pi and other hardware components, as will be discussed in the following sections.

## 2. Materials and Methods

**Figure 1:**
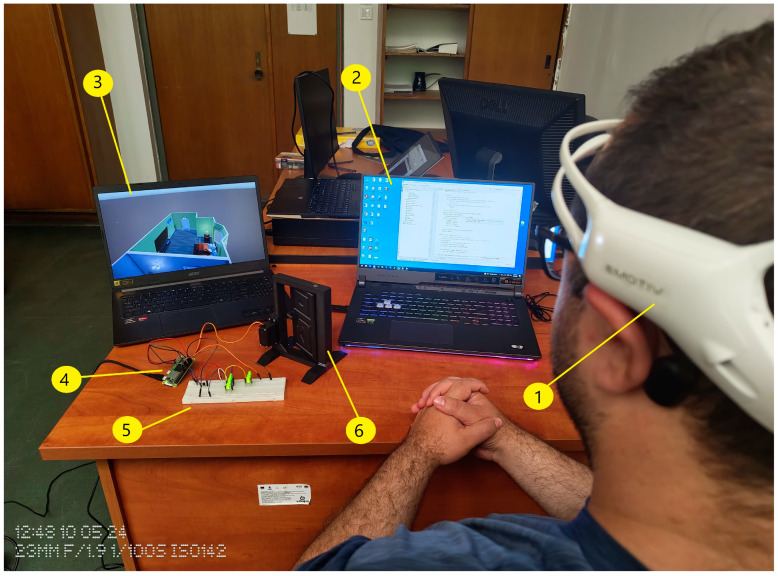
Real-time home automation system and video simulation: 1. EMOTIV Insight neural headset. 2. Cortex API and FastAPI server are running. 3. The simulated system (Unity Engine). 4. Raspberry Pi Zero 2 W. 5. BreadBoard with 5V LEDs. 6. PLA Door and frame, equipped with Digital MG996 servo motor.

The implemented system has been tested in real time, with hardware components have being used to build the system as explained in the following subsections. Also, the system was simulated using Unity Technologies and tested by integrating the EEG headset with the simulated system as explained in Section 2.2.3. Figure 1 shows the system’s components.

**Figure 2:**
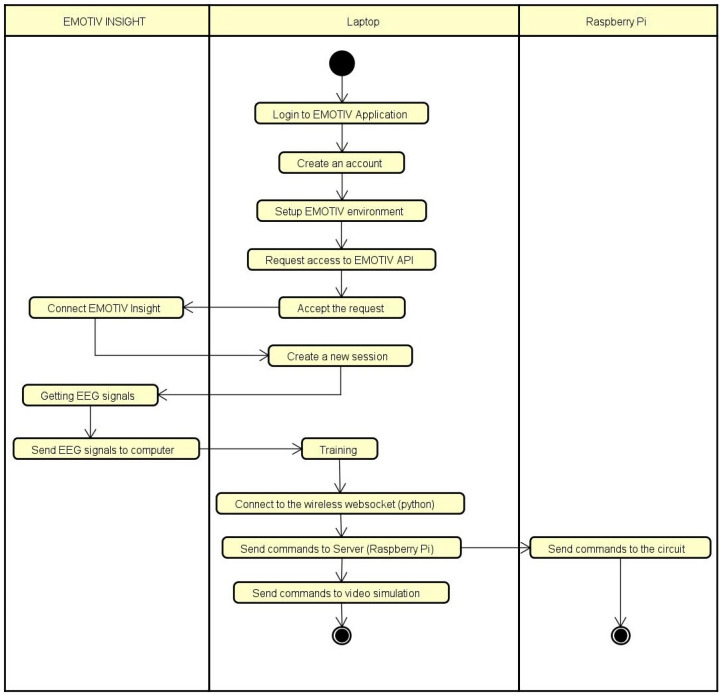
Software activity diagram.

Figure 2 shows the activity diagram of the system’s software.

### 2.1. Material Used for Printing

Polylactic acid (PLA) is a common material used in printing 3D items. This material is biodegradable and produced from renewable sources like maize starch or sugar cane. While it is simple to use in 3D printing without the need for a heated platform, it shrinks in volume as it cools, which is considered a drawback [22]. 

PLA is suitable for many technical applications, particularly in the aviation sector, like prototyping, idea development, and the manufacturing of non-structural elements and interior components. Using PLA in various applications offers benefits like decreased weight, user-friendliness, and cost savings. PLA is unsuitable for applications that involve severe circumstances or exposure to harsh chemicals, like continuous contact with water or marine environments, due to their temperature and environmental constraints [23].

### 2.2. The Technology Used

#### 2.2.1. Technology Used to Design and Print the 3D Door and Frame

The door and frame models of this system were created using CATIA V5-6R2022 software. The models were exported into IdeaMaker Version 5.0.6 software as “stl” files. The resulting models were manufactured using PLA material and a Creality Ender 3 S1 PRO 3D printer (Creality, Shenzhen, China).

Figure 3, Figure 4, Figure 5, Figure 6, Figure 7, Figure 8, Figure 9, Figure 10 and Figure 11 show the 3D print for the door and frame at different stages of printing: 10%, 60%, and 100%.

A calibration of the 3D printer machine must be performed using parameters from Table 1 before starting to print the door and frame.

**Figure 3:**
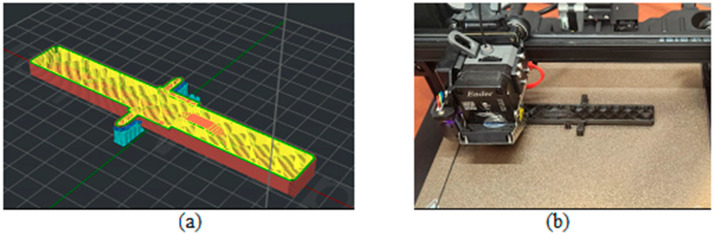
Door model 10%—layer 42: (**a**) software; (**b**) 3D printer.

**Figure 4:**
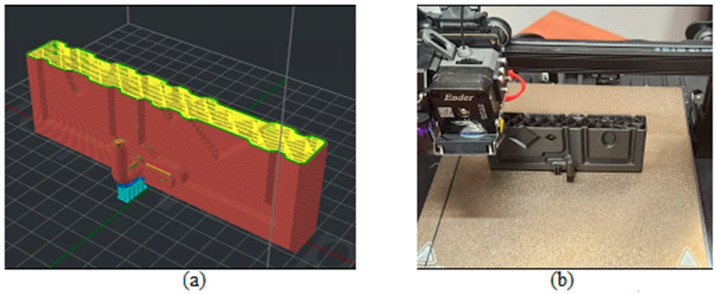
Door model 60%—layer 225: (**a**) software; (**b**) 3D printer.

**Figure 5:**
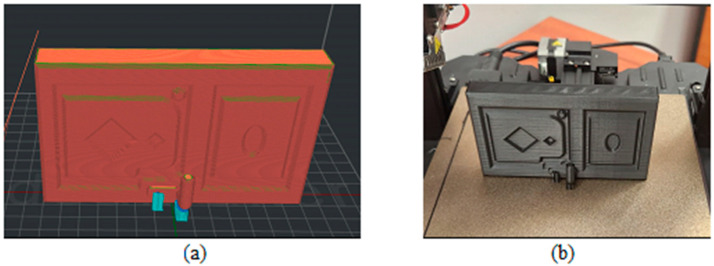
Door model 100%—layer 425: (**a**) software; (**b**) 3D printer.

The door model was printed on 425 layers (Figure 3, Figure 4 and Figure 5).

**Figure 6:**
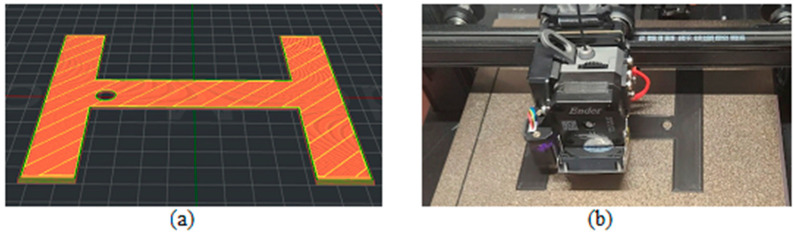
Frame model—first component 10%—layer 5: (**a**) software; (**b**) 3D printer.

**Figure 7:**
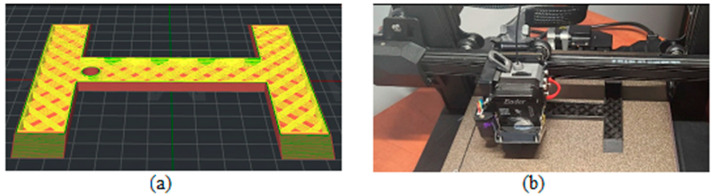
Frame model—first component 60%—layer 30: (**a**) software; (**b**) 3D printer.

**Figure 8:**
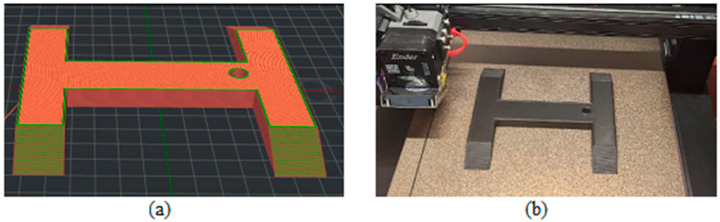
Frame model—first component 100%—layer 50: (**a**) software; (**b**) 3D printer.

**Figure 9:**
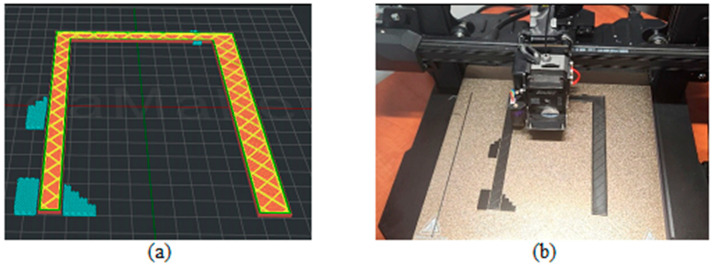
Frame model—first component 10%—layer 12: (**a**) software; (**b**) 3D printer.

**Figure 10:**
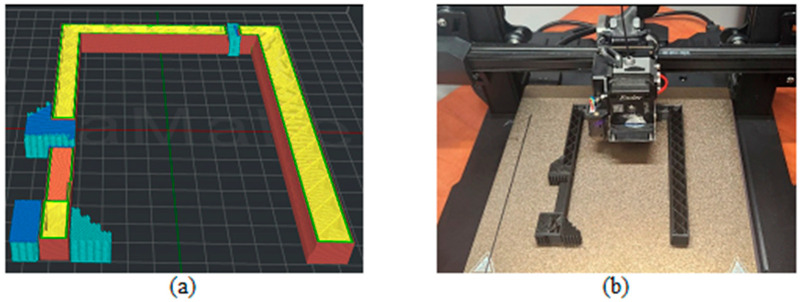
Frame model—first component 60%—layer 72: (**a**) software; (**b**) 3D printer.

**Figure 11:**
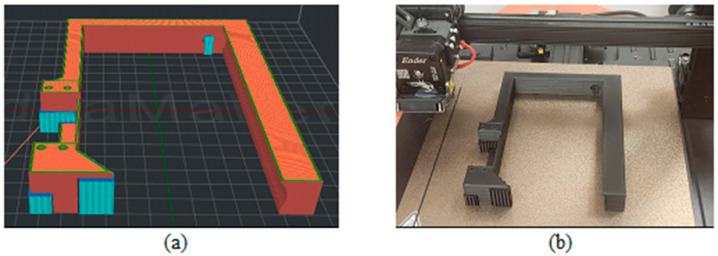
Frame model—first component 100%—layer 120: (**a**) software; (**b**) 3D printer.

The frame model was split into two parts: component 1 was printed in 50 layers (Figure 6, Figure 7 and Figure 8), and component 2 was printed in 120 layers (Figure 9, Figure 10 and Figure 11).

The total cost for the manufacturing of components is around USD 42, and the total printing time is around 17 h, as can be seen in Table 2, Table 3, Table 4 and Table 5.

The implemented system can be considered affordable since its total cost is around USD 420.

#### 2.2.2. The Neural Headset Used as a Tool to Control the Implemented and Simulated Systems

**Figure 12:**
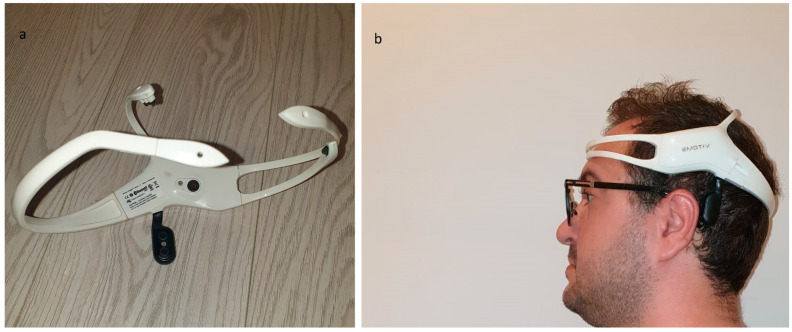
Emotiv™ Insight neuro-headset: (**a**) Semi-dry polymer sensors of the headset; (**b**) the headset on a user head.

This system utilizes the functionality of the EmotivTM Insight headset (Sydney, Hanoi), which is shown in Figure 12 [24]. Five semi-dry polymer sensors and two reference electrodes are part of this five-channel EmotivTM Insight headset. 

The headset’s internal sampling rate is configured at 128 Hz for each individual channel. This cost-effective equipment is specifically designed to facilitate the use of BCIs in experiments and for research purposes. It ensures mobility and high accuracy by filtering brain signals and wirelessly transmitting data to the computer.

The mobile EEG headset has comprehensive brain-sensing capabilities and uses modern electronics to generate high-quality and robust signals. This headset can establish connections with computers and mobile devices via Bluetooth or a 2.4 GHz wireless dongle to transmit the collected raw data to the Processing Unit. Additionally, a LiPo battery with a capacity of 450 mAh has been created to provide eight hours of operation. 

To measure brain activity, it is necessary to position the headset’s electrodes on the scalp as shown in Figure 12.

**Figure 13:**
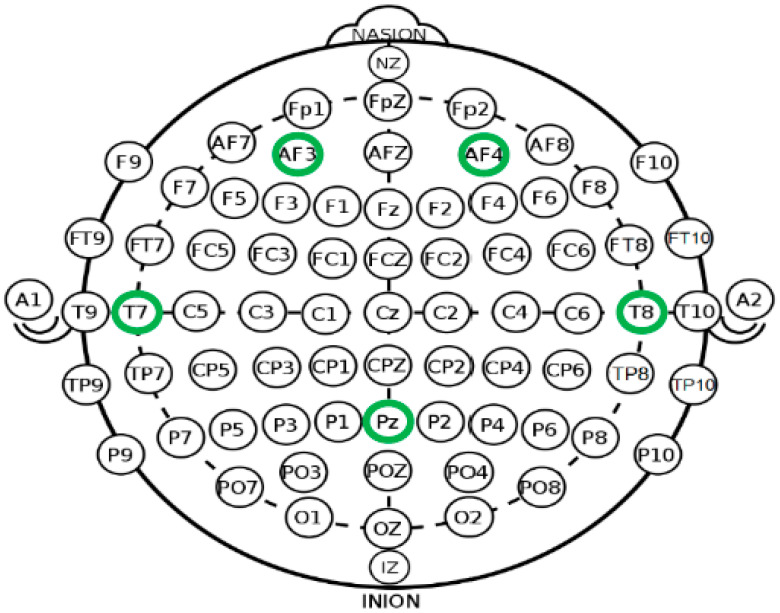
Locations of sensors in Emotiv Insight headset [21].

Figure 13 shows the positioning of the electrodes of the Emotiv headset, including the reference electrodes: DRL (Driven Right Leg) and CMS (Common Mode Sense).

**Figure 14:**
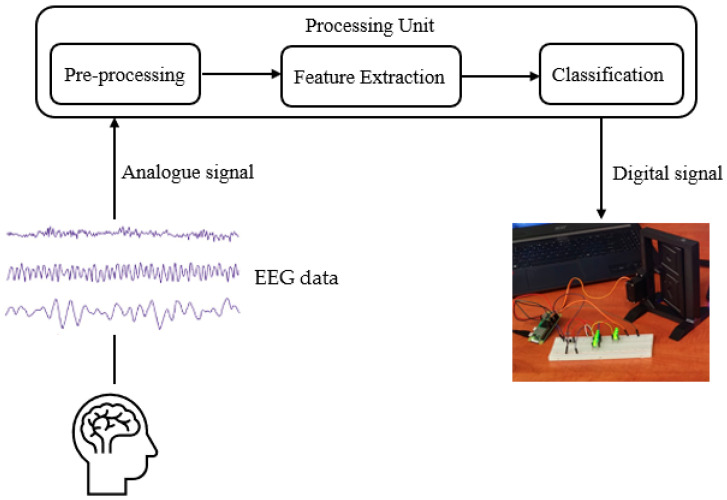
Headset working mechanism.

The headset captures the electrical signal generated by the neurons as an analogue signal [24] (see Figure 14).

The Emotiv Insight headset can amplify the signal and accomplish pre-processing-like signal filtering.

#### 2.2.3. Technology Used in the Video Simulation of the System Operation

Unity is a versatile cross-platform game engine that supports the creation of both three-dimensional and two-dimensional games, as well as simulations. This game engine was first launched in 2005 only for OS X. Unity has been made accessible on a total of 27 platforms, including a wide range of devices like mainstream computer operating systems and major consoles, as well as virtual and mixed reality devices [25,26] Unity has become a very prevalent graphics engine used in the development of virtual simulators of the reality environment [27,28].

**Figure 15:**
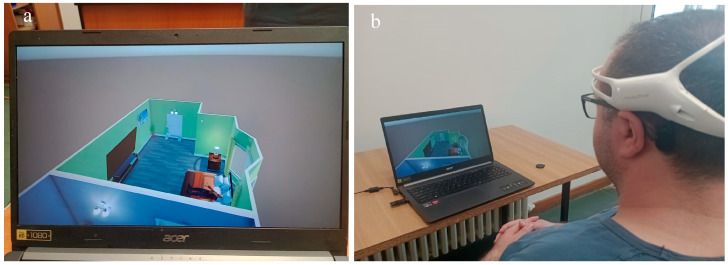
Real-time simulation in Unity using Emotiv Insight: (**a**) simulation of the home view; (**b**) the use of the neuro-headset to control the video simulation.

In the proposed system, the Unity engine is used to simulate a house, where a person controls the lock/unlock of an entry door and turning a light on/off using the Emotiv EEG headset. Actions are triggered by user thoughts through the Emotiv Cortex API (see Figure 15).

### 2.3. Methods

**Figure 16:**
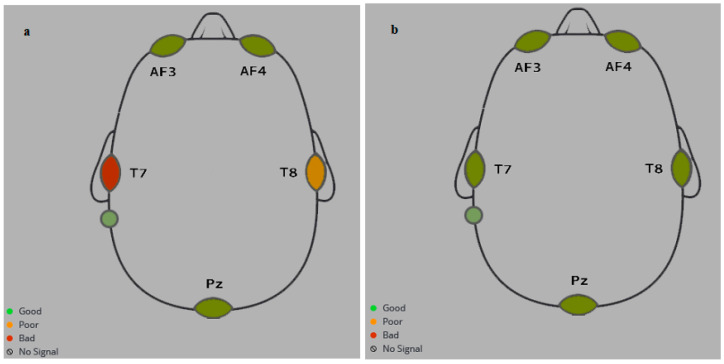
EMOTIV Insight sensors quality: (**a**) bad quality; (**b**) good quality.

**Figure 17:**
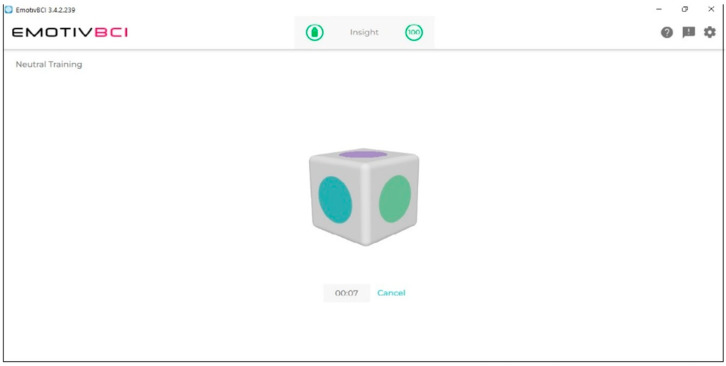
Training session.

The neural headset must be trained by a user to record a thought (a command) for the required control action. It was requested of the participants that they carry out two spoken imagery tasks: turning a power light on and off and opening and closing a door. These tasks will be used later to control the corresponding action. More sessions of training will increase the accuracy of the system in controlling the door and light (see Figure 16). To ensure that all sensors detect high-quality signals, the headset must be adjusted before training (Figure 17).

The training approach included the use of the Emotiv BCI application, where each command was trained for 100 training iterations (8 s for each iteration). The total time of the training session was around 25 min.

#### 2.3.1. System Implementation

**Figure 18:**
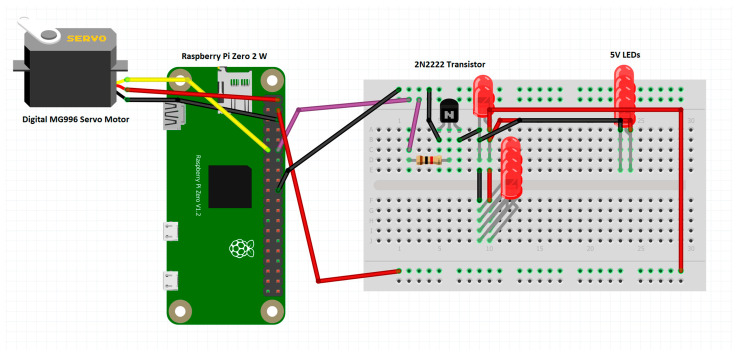
Circuit diagram made with Fritzing [33].

The system comprises multiple components connected as follows: the Emotiv Insight neuro-headset establishes a Bluetooth connection with the computer, the computer establishes a Secure Shell Protocol (SSH) connection with the Raspberry Pi Zero 2 W, and the Raspberry Pi is connected to the Digital MG996 servo motor and 10 LE’s via the GPIO pins (see Figure 18).

The Raspberry Pi Zero 2 W (Pencoed, UK) [29] is a tiny, powerful single-board computer developed by the Raspberry Pi Foundation. It contains a 1 GHz ARM Cortex-A53 quad-core processor; it offers solid performance in a compact package. With integrated Wi-Fi (802.11b/g/n) and Bluetooth 4.2 connectivity, it enables wireless communication for networking and peripheral connections. The hardware offers a performance of approximately 80% of that of the Raspberry Pi 3B and is five times faster than the original Raspberry Pi Zero. Its small size and versatile features, including GPIO pins for hardware communication like with the servo motor and a microSD card slot for storage, make this model ideal for IoT applications and projects that require a compact size and low cost.

The Digital MG996 servo motor (SHTM, Hong Kong, China) [30] is a high-torque component with precise 90° movement, ideal for robotics and remote control applications. Its digital control interface ensures accuracy and reliability, while its robust construction and metal gears provide durability. Perfect for projects requiring precise angular control in a compact package, it is suitable for this system.

Many low-power amplifying and switching applications make use of the 2N2222 [31], a popular NPN bipolar junction transistor (BJT). It is good for our 10 LEDs that operate at 5V and would draw in a total of around 200 mA.

The LED used in this system is L-7113GD-5V. It is a 5 mm green LED [32] with a water-clear lens, a wide viewing angle of approximately 60 degrees, a wavelength of 565 nm, a forward voltage of 5V, and a typical brightness of 15-30 millicandela. It is ideal for our application as it also has an internal resistor, thus minimizing the amount of wires and other components used.

One of the 5 V GPIO pins, a ground pin, and the GPIO 17 Chip Enable-CE1 (SPI1) pin of the Raspberry Pi Zero 2 W are connected to the VCC pin, ground pin, and PWM pin of the servo motor. The GPIO 18 Chip Enable-CE1 (SPI1) pin is connected through a 1kΩ resistor to the 2N2222 transistor (SHTM, Hong Kong, China), and the emitter and the collector are connected to the ground of the RPi, and the other 5V GPIO pin of the Pi is the VCC pin. We connect 10 of the 5V LEDs with internal resistors for lighting.

#### 2.3.2. System Control

The filtered brain signal is sent from the headset to the computer by Bluetooth and will be converted into specific commands using CortexAPI to be run by the Raspberry Pi Zero 2 W. The Cortex API represents an interface created by Emotiv to manage EEG data captured by the headset. The resulting commands are sent to the Raspberry Pi Zero 2 W by SSH (Secure Shell Protocol) to execute two Python scripts. The Python script is running to open/close the door and turn the LED on or off depending on the commands received. For example, to open or close the door, the user thinks of the corresponding speech imagery word that he/she thought in the training sessions.

A live server is set up on the platform using Python and the FastAPI framework. As shown in Algorithm 1, updating the Cortex API library is necessary to increase control over the headset. These modifications reduce the latency of the orders sent and improve the headset’s connection. Additionally, these updates facilitate subscribing and receiving commands sent to a WebSocket address.

After transforming the signals to the server, there is a predefined brief delay to guarantee the successful transmission of the signal. The commands are processed and organised using a dictionary structure, in which each command corresponds to a certain action (Algorithm 1). For instance, when the specific command is executed, it produces a JSON response. Once the answer is organised, it is efficiently sent using a separate WebSocket that is particularly created for the purpose of transporting instructions to apps and receiving replies from applications to the server. 

Responses are received on the Commands WebSocket route at the application layer. After receiving a command, the application decodes it and assigns it to the appropriate action.
**Algorithm 1** Cortex API updates in Python1: Define class ConnectionManager 2:     Define initialisation 3:         Set active_connections as an empty list of WebSockets 4: 5:     Define method Broadcast 6:         If the time since last_sent exceeds 3 s 7:             Send a text message to all active_connections 8:             Update last_sent_time 9:             Log message sent 10:        Else 11:            Log message sending skipped due to cooldown 12: 13: Define WebSocket Endpoint “/ws” 14:     Connect incoming WebSocket 15:     Continuously receive text from WebSocket 16:     On exception, log and disconnect WebSocket 17: 18: Define Background Task to Listen for Commands 19:     Continuously 20:         Retrieve data from the Cortex instance 21:         If valid command data received with sufficient power 22:             Process and broadcast command 23:         Handle exceptions with error logging and retry with delays 24: 25: Define Command Processing 26:     Depending on the command 27:         Set descriptions for recognised commands 28:         Convert command and description to JSON 29:         Broadcast JSON message 30: 31: Define Application Startup Event 32:     Initialise and configure the Cortex instance 33:     Connect and authorise with Cortex 34:     Create a session and subscribe to the mental commands data stream 35:     Start background task for listening to commands

**Figure 19:**
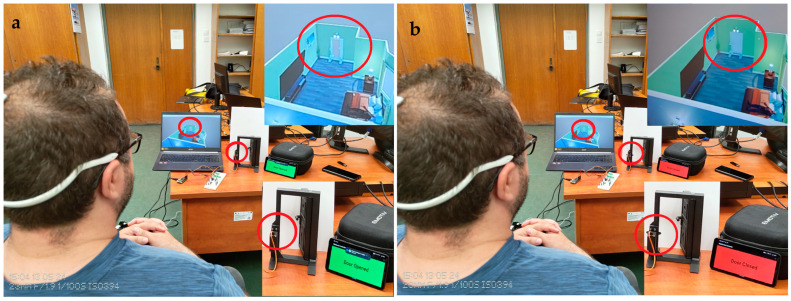
Lock and unlock the door using brain signals, with real-time notifications on video simulation and phone: (**a**). Door unlocked; (**b**). door locked.

**Figure 20:**
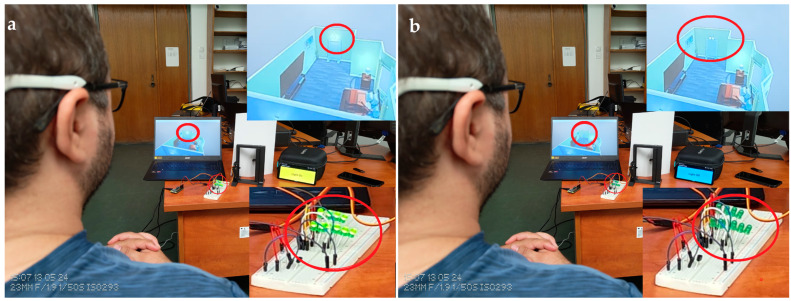
Changing LED ON/OFF using brain signals, with real-time notifications on video simulation and the phone: (**a**). LED ON; (**b**). LED OFF.

A Flutter application has been developed for real-life use and to check the door status. The latest version of Flutter (3.19.5) is used to ensure maximum compatibility with both Android and iOS. This application listens to the WebSocket server running on the Raspberry Pi Zero 2 W IP, which can be chosen using the settings of the app (default being “localhost”). When the door or the light status changes (e.g., opening the door), the server sends a signal to the Flutter application WebSocket client. As can be seen in the Algorithm 2 functions, the application can display what is currently happening. When the app receives a signal to open the door from the server, a “Door Opened” message is displayed on the screen along with changing the background of the app to green for better demonstration, and when the app receives the same signal again, the message “Door Closed” is displayed and the background colour is changed to red (see Figure 19). Similarly, when the server sends a signal to turn the light on, a “Light On” message appears on the screen and the background colour of the application is changed to yellow. When the same signal is received again, the message “Light Off” is displayed and the background colour of the application is changed to grey (see Figure 20). As the application is developed in Flutter, it can be run on all platforms, including Android, iOS, and Windows, on which it has been tested and confirmed to be working properly.
**Algorithm 2** Functions developed in Flutter, to be run on the phone1: FUNCTION connectToWebSocket 2:    SET channel TO IOWebSocketChannel.connect(Uri.parse(‘ws://$ipAddress:8000/ws’)) 3:    SET channel.stream LISTEN processMessage 4: END FUNCTION 5: 6: FUNCTION processMessage(message AS STRING) 7:     DECLARE decodedMessage AS OBJECT 8:     SET decodedMessage TO jsonDecode(message) 9:     DECLARE command AS STRING 10:    SET command TO decodedMessage[‘command’] 11:     12:     IF command EQUALS ‘door action corresponding word’ THEN 13:         INCREMENT pushCount BY 1 14:         SET displayMessage TO IF pushCount MOD 2 EQUALS 1 THEN ‘Door Opened’ ELSE ‘Door Closed’ 15:         SET backgroundColor TO IF pushCount MOD 2 EQUALS 1 THEN Colors.green ELSE Colors.red 16:     ELSE IF command EQUALS ‘light action corresponding word’ THEN 17:         INCREMENT pullCount BY 1 18:         SET displayMessage TO IF pullCount MOD 2 EQUALS 1 THEN ‘Light On’ ELSE ‘Light Off’ 19:         SET backgroundColor TO IF pullCount MOD 2 EQUALS 1 THEN Colors.yellow ELSE Colors.blueGrey 20:     END IF 21:     CALL setState 22: END FUNCTION

#### 2.3.3. Demonstrative Real-Time Unity Simulation of Controlling Home Automation System Using BCI

To simulate a home automation system controlled by the user’s mind, the Unity engine has been used to create a real-time video simulation of the residential environment. This simulation comprises a meticulously designed 3D model of a house inside the engine, integrated with a specialised neural headset used to record the participant’s thoughts. The simulation includes a three-dimensional representation of a door to achieve a high level of realism in the scenario. An essential component of the simulation is the use of the NativeWebSocket library, which facilitates the establishment of a connection between the simulation and the server (Algorithm 3).
**Algorithm 3** WebSocketController class in C#1: CLASS WebSocketController EXTENDS MonoBehaviour 2: 3:     FUNCTION Start 4:         CREATE WebSocket to “ws://localhost:8000/ws” 5:         SET event handlers for WebSocket (OnOpen, OnError, OnClose, OnMessage) 6:         CONNECT to WebSocket 7:     END FUNCTION 8: 9:      FUNCTION Update 10:         IF NOT running in WebGL OR running in Unity Editor THEN 11:             DISPATCH message queue from WebSocket 12:         END IF 13:     END FUNCTION 14: 15:     FUNCTION OnApplicationQuit 16:         CLOSE WebSocket 17:     END FUNCTION 18: 19:     FUNCTION OnMessage(bytes AS Byte Array) 20:         CONVERT bytes to messageJson STRING 21:         PARSE messageJson to CommandMessage 22:         IF mental command is “‘door action corresponding word’ “ THEN 23:             START Flashing to toggle door based on door’s current state 24:         ELSE IF mental command is “light action corresponding word’ “ THEN 25:             START Flashing to toggle lights 26:         END IF 27:     END FUNCTION 28: 29:     FUNCTION Reconnect 30:         CONNECT to WebSocket again 31:     END FUNCTION 32: 33: END CLASS

The WebSocket protocol reduces the latency in command execution. When the server sends a command, the system carries out certain tasks that have been assigned to each command.

To enhance the realism, physical forces at specific points of the door were simulated, creating the action of a servo motor in practical applications. This included the detailed realism of opening and closing the door.

**Figure 21:**
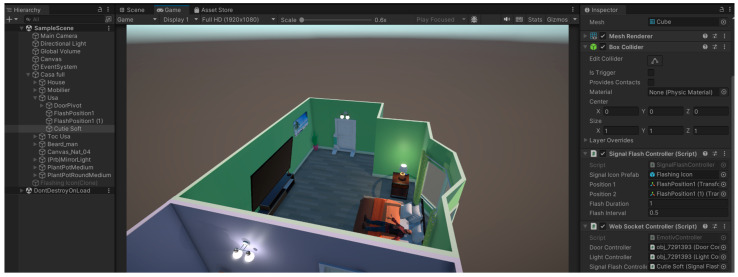
Home automation system simulated with Unity engine.

In addition to door mechanics, the simulation extended to a lighting system in the house model. Also, the door light is simulated to be switched on and off via commands using the headset. The aspect of the simulation using the Unity engine capabilities to propagate light from a source illuminates the 3D space in a manner consistent with the real world (see Figure 21).

## 3. Results and Discussion

Twenty people participated in the study by having their EEG signals recorded. The proposed system was tested on healthy people, since it is tough to test on disabled people. The participants were 16 males and 4 females, with ages between 19 and 34. To control the video simulation and the implemented system, there were two commands on the EMOTIV Insight neural headset for each participant. Every participant was required to put on the dedicated neural headset and take a seat in front of the computer (for the simulation) and in front of the implemented hardware system (see Figure 19 and Figure 20). 

Initially, each participant was instructed to activate the light by mentally focusing on the specific image or phrase that they had been trained to associate with it. Once the light was activated, the individual was instructed to deactivate the light. Similarly, the participant was asked to open and close the door. Each participant was instructed to attempt to manipulate the light and door fifteen times for each order and to keep track of the number of instructions that were successfully executed (the attempts that resulted in the successful control of the light and door). To execute the required Python file, the user’s PC sends a command through Bluetooth to the Raspberry Pi whenever they think about one of these instructions. This script includes the orders to transmit the appropriate signal to the light and door. All these changes are notified in real-time on a localhost server, which sends the notification to a Futter application developed to be used on the phone.

The calculation of the standard deviation (STDEV) entails computing the square root of a number obtained by comparing individual data points to the overall mean of a defined population. To calculate STDEV for each participant from Table 6, Microsoft Excel as statistical software has been used.

Table 6 displays the quantity of successfully executed commands for each participant during the trial. The examination of the results showed that a lack of concentration throughout the experiment could have contributed to some of the commands executed. Another possible explanation for the somewhat better success rate of male replies compared to female responses is that the EEG signal quality was marginally worse in certain female individuals owing to their longer hair. A more accurate EEG headset with better sensing and more electrodes can be used to overcome this issue.

The overall average is 10.52, although the examination indicates that the overall precision of the system is 70.16%. The dispersion has been determined by calculating the standard deviation for each set of data separately for each participant. The highest and lowest values obtained were 1.41 and 0, respectively. The experiment often has a low standard deviation, with an average standard deviation of 0.8131 for all results. Dispersion is seen in participants 4, 6, 13, and 14 as a result of a variation in the relevance of the success attempts for the two trained commands.

Mental commands sent from the neural headset have successfully controlled the video simulation and the physical system in parallel, both versions being synchronised to give a response in a similar time as can be seen in Figure 19 and Figure 20.

The participants reported that the headset became uncomfortable after 20 min. Most of them said they could easily control the light and the door.

## 4. Limitation

Our proposed system is just a prototype for research purposes. To improve the system, a more accurate, non-invasive EEG headset with better sensing and more electrodes can be used. Also, a semi-invasive or invasive BCI can be used, which requires a surgical invasion.

Bluetooth connections cannot interfere with other signals, like Wi-Fi signals in the house. Emotiv Insight headsets can reduce or eliminate noise that can be generated by interferences. Also, the signal quality of the headset can be affected by the thick hair of a user.

## 5. Conclusions

In the realm of biomedicine, the applications of BCI applications that are based on EEG are the intended objective of the present research. The objective of this study is to develop an implementation for the execution of a real-time home automation system that is controlled by brain signals. Patients with quadriplegia, locked-in syndrome, and other conditions that prevent them from walking or moving may utilize the device, as can healthy persons who want to live a more comfortable life. A further benefit of this work is that it paves the way for the control of a greater number of household appliances via the use of BCIs.

Table 7 shows a comparison between the proposed implemented system and related works.

When compared to the performance of the other studies that were provided in the literature study, the BCI system that is presented displays similar results, and the following observations can be made referring to this implemented system: it contained a video simulation and a connection to a real-time server to send live notifications to smartphones, and the physical system was successfully tested on twenty participants.

For future work, the presented system can be upgraded to fulfil other tasks than the current ones, but for every task, a new neural command must be trained by the user who wears the headset. Semi-invasive or invasive BCIs can be used also, which requires a surgical invasion. Also, techniques like System on a Chip (SoC) should be used.

## Figures and Tables

**Table 1:** Printing parameters.

Field	Value
Layer height	0.2 mm
Layers on contour	2.5
Filling density	5%
The type of filling	Straight
Printing plate temperature	60 °C
Printing head temperature	210 °C

**Table 2:** Time, quantity, and estimated price for the door 3D printing.

Description	Value
3D printing time	8 h, 15 min, and 50 s
Amount of material used [g]	81.9
Estimated price [USD]	19.49

**Table 3:** Time, quantity, and estimated price for the frame 3D printing—the first component.

Description	Value
3D printing time	2 h, 52 min, and 42 s
Amount of material used [g]	25.9
Estimated price [USD]	6.16

**Table 4:** Time, quantity, and estimated price for the frame 3D printing—the second component.

Description	Value
3D printing time	5 h, and 29 min
Amount of material used [g]	52.6
Estimated price [USD]	12.53

**Table 5:** Total time, quantity, and estimated price for the door and frame 3D printing.

Description	Value
3D printing time	16 h, 37 min, and 32 s
Amount of material used [g]	175.8
Estimated price [USD]	41.84

**Table 6:** Testing results.

Participants	Light On/Off	Door Locked/Unlocked	AVG	STDEV
1	11	12	11.5	0.7071
2	9	10	9.5	0.7071
3	8	7	7.5	0.7071
4	12	10	11	1.4142
5	9	10	9.5	0.7071
6	11	9	10	1.4142
7	10	11	10.5	0.7071
8	12	13	12.5	0.7071
9	13	14	13.5	0.7071
10	11	10	10.5	0.7071
11	12	12	12	0
12	7	8	7.5	0.7071
13	9	7	8	1.4142
14	11	9	10	1.4142
15	9	10	9.5	0.7071
16	13	12	12.5	0.7071
17	11	10	10.5	0.7071
18	10	11	10.5	0.7071
19	12	11	11.5	0.7071
20	12	13	12.5	0.7071
Total AVG			10.525	
STDEV AVG				0.8131

**Table 7:** Comparation with related works.

Related Work	Advantages	Disadvantages	How This System Is Different
Controlling of Smart Home System Based on Brain–Computer Interface	- Accurate, EMOTIV Epoc has been used - Four commands are available	- Expensive - Complex	- Affordable - Door control - Real-time notifications
Brain–Computer Interface-Based Arduino Home Automation System for Physically Challenged	- Affordable	- Not accurate - Only one command (when blinking)	- Two commands - More accurate - Door control - Real-time notifications
Electroencephalography (EEG)-Based Home Automation for Physically Challenged People using Brain–Computer Interface (BCI)	- Affordable	- Not accurate - Only one command (when blinking)	- Two commands - More accurate - Door control - Real-time notifications
IoBCT: A Brain–Computer Interface using EEG Signals for Controlling IoT Devices	- Accurate - Affordable	- Only one command	- Two commands - Door control - Real-time notifications
Enhancing Home Automation through Brain–Computer Interface Technology	- Accurate, EMOTIV Epoc has been used	- Expensive	- Door control - Real-time notifications

## Data Availability

No new data were created or analysed in this study. Data sharing is not applicable to this article.
